# *Haemophilus parahaemolyticus* Septic Shock after Aspiration Pneumonia, France

**DOI:** 10.3201/eid1910.130608

**Published:** 2013-10

**Authors:** Anne-Sophie Le Floch, Nadim Cassir, Sami Hraiech, Christophe Guervilly, Laurent Papazian, Jean-Marc Rolain

**Affiliations:** L'Institut Hospitalier Universitaire Méditerranée Infection, Marseille, France

**Keywords:** *Haemophilus parahaemolyticus*, pneumonia, emerging pathogen, matrix-assisted laser desorption ionization time-of-flight, MALDI-TOF, respiratory infections, bacteria, France

**To the Editor:** Members of the genus *Haemophilus* are commensal bacteria of the upper respiratory tract, and *H. influenzae* is the main pathogen in this genus that can cause a wide range of human infections (*1*). The species most closely related to *H. influenzae* is *H. haemolyticus*, usually considered a commensal of the nasopharynx in humans; it can be pathogenic, although rarely (*2*,*3*). *H. parahaemolyticus* was distinguished from *H. haemolyticus* in 1953 when it was determined that *H. parahaemolyticus* required only factor V, but not factor X, for growth (*4*). This species could be responsible for pharyngitis and, rarely, for subacute endocarditis (*4*), but it has seldom been reported to cause invasive disease (*5*). Invasive disease has been reported in a patient who had an empyema in the gallbladder (*6*) and in a patient who had a cryptogenic brain abscess (*7*). We report a case of acute respiratory distress syndrome (ARDS) and septic shock caused by *H. parahaemolyticus*.

A 50-year-old woman, who was receiving artificial ventilation, was transferred to the Hôpital Nord in Marseille, France, in November 2012. She was in a coma because she had taken an overdose of benzodiazepine and clomipramine in a suicide attempt. She had no relevant medical history except addiction to tobacco, chronic alcoholism, and depression. The patient’s family owned 3 cats and 1 dog but had no other pets. She had not traveled outside France.

Notable findings on initial examination (before intubation) were shock (arterial blood pressure 80/50 mm Hg, despite adequate fluid resuscitation), tachycardia (pulse rate 110 beats/min), tachypnea (respiratory rate 34 respirations/min), low peripheral oxygen saturation (S_p_O_2_ 80%), and a temperature of 39°C. On intensive care unit (ICU) admission, her partial pressure of arterial oxygen/fraction of inspired oxygen ratio was 101 under mechanical ventilation, with a positive end-expiratory pressure level of 10 cm H_2_O, consistent with ARDS. Chest examination revealed bilateral crackles on lower lung lobes, consistent with chest radiograph findings. Routine laboratory evaluation showed a hemoglobin level of 11.7 g/dL (reference 12.5–15.5 g/dL for women); leukocytosis, indicated by a leukocyte count of 23 cells × 10^9^/L (reference 4–10 ×10^9^/L); and elevated C-reactive protein level (298 mg/L [reference <5 mg/L]).

On the second day, a bronchoalveolar lavage (BAL) specimen (obtained before initiation of antimicrobial drug therapy) was positive for *H. parahaemolyticus* (10^7^ CFU/mL) on a chocolate polyvitex agar plate, and the strain showed susceptibility to ampicillin/clavulanate, ceftriaxone, rifampin, gentamicin, and ciprofloxacin. The bacterium was hemolytic and required factor V, but not factor X, for growth. The bacterium was identified in the laboratory by matrix-assisted laser desorption ionization time-of-flight (MALDI-TOF) mass spectrometry with the Bruker Biotyper software database (Bruker Daltonics, Bremen, Germany), with a good score (>2.0) (*8*). The identification was confirmed by PCR amplification and sequencing of the 16S rRNA gene (size of sequence was 1,387 bp, and it had 99.6% homology with sequence AJ295746 in GenBank). Blood culture results were negative for both bacterial species. The final diagnosis was septic shock associated with ARDS, due to aspiration pneumonia.

In the ICU, the patient received ampicillin/clavulanate and gentamicin, along with vasopressor therapy and crystalloid fluid resuscitation. Her response was dramatic, and her condition improved rapidly. When she was stabilized and able to take oral drugs, she was given ampicillin/clavulanate, 1 g 3× daily for 7 days. On day 9, she was discharged from the ICU in stable condition. One month after discharge, she attended a follow-up visit at the pulmonary outpatient department and had made a full recovery.

This study shows the isolation in pure culture of *H. parahaemolyticus* from the BAL specimen of a patient with septic shock with ARDS. The isolate was unambiguously identified by MALDI-TOF (*8*) and confirmed by 16S rRNA sequencing. Correct identification of bacteria of the genus *Haemophilus* at the species level, including *H. parahaemolyticus*, by MALDI-TOF, has also been reported in 2 recent works (*9*,*10*). Isolation of this bacterium in pure culture from the BAL specimen was eventually associated with the disease of the patient (including a coma complicated with aspiration pneumonia and bilateral pulmonary consolidations), and the patient rapidly improved after receiving antimicrobial drug treatment.

These findings suggest that *H. parahaemolyticus* was the causative agent of the patient’s disease. Although this bacterium has been rarely reported as a cause of human infections, it should be considered as an opportunistic pathogen, especially in patients who have aspiration pneumonia, because it is likely a commensal of the upper respiratory tract. Among 31 *H. parahaemolyticus* isolates from human specimens reported by Nørskov-Lauritsen et al., 75% of the isolates were recovered as commensals in the pharynx and throat and from sputum (*10*). This bacterium was likely overlooked in the past because phenotypic identification was not sufficiently accurate to distinguish it from other *Haemophilus* spp. Thus, *H. parahaemolyticus* has a pathogenic potential for causing invasive and severe diseases in humans that should be further investigated.
